# Looking into the past to build the future: food, memory, and identity in the indigenous societies of Puebla, Mexico

**DOI:** 10.1186/s42779-022-00123-w

**Published:** 2022-02-23

**Authors:** Diosey Ramon Lugo-Morin

**Affiliations:** Postgraduate Program in Sustainable Management of Natural Resources, Management of Socioecological Systems, Research and Postgraduate Studies, Intercultural University of Puebla State, Calle Principal a Lipuntahuaca S/N. Lipuntahuaca, C.P. 73475 Huehuetla, Puebla Mexico

**Keywords:** Bioculture, Indigenous, Memory, Traditional foods, COVID-19, Quelites, Wild plants

## Abstract

Food memory has been determinant in the survival of social groups, the causes of its activation are varied (e.g. feelings, identity, need or challenge) and it is transmitted from generation to generation. These interactions have given rise to an ethnic food heritage that responds to cultural processes and territorial specificities. It is in this logic that this study is proposed, which aims to explore the dynamics between food, memory, and identity of two ethnic groups in the state of Puebla in Mexico in a context of health disruption. The experience of these two groups shows how food identity and memory is particularly strategic in the face of adversity, as is currently the case with the COVID-19 pandemic.

**Supplementary Information:**

The online version contains supplementary material available at 10.1186/s42779-022-00123-w.

## Introduction

This study proposes to explore the dynamics between food, memory and identity of two ethnic groups in the Puebla state in Mexico in a context of health disruption. Food shortages were present before and after the prehispanic period [1], and these events were recorded in the Codex Chimalpopoca [2] and the “Relaciones Geograficas del Nuevo Mundo” [3]. In the aforementioned events, the region of the Sierra Norte de Puebla played a strategic role due to the perpetuity of the food systems, which its inhabitants developed in a context of high biodiversity; the prehispanic famine [4] and the food shortages in colonial times [5], determining elements in the biocultural food configuration of the main ethnic groups settled in the Sierra Norte de Puebla, as well as in other ethnic regions. According to Morell-Hart [6], changes from cultivated to wild plants can be observed in paleoethnobotanical samples, and these dietary dynamics may constitute evidence of a response strategy in the face of a famine.

The indigenous treatment of the nascent food systems incorporated a strong influence of their worldview, allowing for new processes of experimentation and learning in the context of the existing plant biodiversity of the new world [7], this food bioculture was forged before the arrival of the Spanish in the New World and after their arrival new cultural elements were added. The capacity for indigenous agency was invaluable in overcoming prehispanic famine, and to date, this capacity remains unchanged; some examples in Mexico and Europe have been recorded and documented [8–11].

Food bioculture is extensive and more complex than is widely believed [12, 13] globally over 6000 indigenous groups are evidence of this reality [14]. Its potential is limitless and has enabled indigenous communities to cope with past challenges (e.g. pandemics) [9, 15].

Mexico is part of this reality; in its territories, we find 11 linguistic families in 68 ethnic groups. This fact shows a cultural richness that places this country as one of the ten most linguistically diverse nations in the world [16]. In this context is located the Sierra Norte de Puebla, a vibrant region in central Mexico that is home to seven indigenous groups: totonac, ngigua, nahua, hñähñu, tepehua, mazatec and mixtec, each group possessing a cultural richness that is manifested in rituals, ceremonies, foods and worldviews derived from their prehispanic and colonial past. According to Maffi [17], the concept of biocultural diversity expresses a holistic idea of the diversity of life in nature and culture. It considers biodiversity, cultural diversity, and linguistic diversity as interrelated and interdependent manifestations of the net of life.

**Fig. 1:**
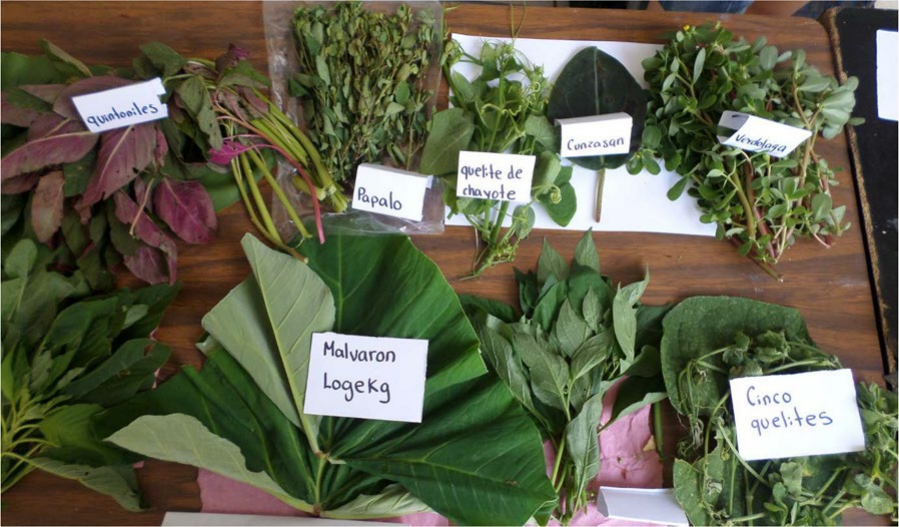
Sample of some of the edible wild plants (Quelites). These plants are present throughout the year in the region and are important in the ethnic diet of indigenous groups

The phytogenetic resources of the Sierra Norte de Puebla region share an origin with Mesoamerica, constituting 15.4% of the species of the world food system (1500 edible plants are located in the Mesoamerican cultural region, of which 182 are from the Sierra) (*see* Fig. 1), which gives rise to an important biocultural richness [16, 18–20].

This provides the region and its people with an ethnic food heritage that includes a diversity of diets and edible plants (known in the region as quelites); through this study, we will present some of these diets, their preparation and plants used. This diversity of edible plants is distributed in indigenous territories through a network of local markets that constitute the phytogenetic bank of indigenous peoples [16, 21, 22], which has allowed indigenous people to preserve several original genetic lines in their territories, marking the relevance of ethnic bioculture [16, 23].

### Food and ethnic identity: relationship and scope

Food has been a human need since humans appeared on Earth. The ways in which humans eat have been mediated by their cultural identity and the territory in which they live. It is to be expected that each social group will have food and eating habits that are differentiated by the specificities of their territories [24].

Food habits determine the frequency and intensity of consumption of certain foods and flavours, but also the configuration of these habits can be shaped by adverse events at the micro-, meso- or macro-level [25]. Ethnic foods have persisted over time linked to their territories, and recent studies argue that this food group has been able to adapt to the preferences and needs of a variety of consumers from different cultures [26, 27]. A study in Mayan territory argues that the persistence of these foods historically is associated with access, power relations, modern responses for an edible plant-based diet is growing, and in the West needs is reflected in a diet with higher inputs of green ingredients [28, 29]. Indeed, ethnic foods have gained prominence in modern civilization.

The appropriation of this ethnic food legacy has rested in recent centuries on the memory of indigenous people. The memory of the human species is genetic, linguistic, and cognitive [30, 31], and is expressed in the diversity of genes, languages and knowledge or wisdom [32]. This attribute allows the human species to look to the past to build a possible future. How would such a future be possible? Of course, by revealing the relationships that humanity has established with nature throughout history [33].

Humans persist in a variety of ecological environments, displaying an enormous diversity of behaviours [31, 34]. This has led them to develop coping strategies that have enabled their survival, and old and new challenges (e.g. climate change, pandemics, food insecurity) have led them to reassess their actions, for which biocultural memory, among other elements, is fundamental [35].

Globally, biocultural memory is represented by a “hard-core” of indigenous peoples numbering 476 million people [14, 36, 37]. According to Toledo [38] and Price [39], cultural diversity has generated differentiated contexts that have promoted learning, experimentation, and memorization of diverse relationships with nature.

According to Karim-Aly [40] knowledge is networked with idea and action. This takes on significance in research because science and practice are not separated. If theory and practice are divorced, they risk becoming invisible because they are disconnected from their context. This is especially true if we are talking about cultures with a strong connection to their environment.

The construction of an ethnic food knowledge has been made possible by the connections between culture and nature in a context of changes and mobilizations that indigenous people have historically capitalized on in a food bioculture [7, 41–43]. Food bioculture is defined as biodiversity humanized from ancestral times by indigenous groups. The interaction between indigenous people and nature has enabled a balanced and respectful social metabolism, which continues to this day [44].

The prehispanic and colonial history of indigenous people in Mexico has not been static but dynamic, trade routes or food shortage events are recorded facts [2–5, 37, 45, 46]. Mexico as megadiverse country has promoted the construction of a bioculture [47, 48].

In other ethnic regions of the world, we can identity these logics in the context of changes and mobilizations that make biocultural heritage possible, for example, the indigenous Warao, who have inhabited the Orinoco Delta in Venezuela for more than 2,000 years [49]. On the other hand, the indigenous people of the central Kalahari are estimated to be 18,000 years old [50]. In Australia, indigenous people have reached an age of over 50,000 years [51, 52], and Pygmies have lived in African forests for 60,000 years [53, 54]. Despite the resilience of indigenous people, we seem to have learned nothing; in less than three centuries, modern civilization has brought the planet to the brink of collapse [55, 56]. This analysis aims to show, through ethnic food memory, how informal institutions are important in the configuration of a new food reality that allows the revaluation of food heritage in the face of challenging scenarios such as climate change, food insecurity or pandemics.

### Biocultural food memory

Biocultural food memory reveals the different interrelationships that a social group has with nature in the process of configuring its diet. The need to feed themselves has led indigenous groups since time immemorial to explore, learn about and experiment with nature, and these processes have provided them with extensive ancestral knowledge of the flora of the territories they have dominated.

But when we talk about food memory, we also evoke sensations and consider memories as an inseparable part of time [57]. This way of exploring food memory transports us from the past to the present and endows us with a prospective memory. According to [58], prospective memory is omnipresent in our daily lives. We rely on this type of memory to strengthen our resilience, an example of which was the emergence of the COVID-19 pandemic, where most indigenous people remembered the edible plants used by their ancestors to cope with different diseases [9]. In this sense, some indigenous communities in the Sierra Norte de Puebla have adopted this coping strategy that their ancestors used in the face of adversity, particularly health.

The characteristics of an ethnic diet are based on the worldview of the indigenous group, thus indigenous people have been able to identity plants for different uses in correlation with their needs. The indigenous groups memory records with each plant are kept by individual elders of the group and transferred from generation to generation through oral tradition. One example is Mafafa (*Xanthosoma robustum*), a wild plant that was used by indigenous nahua elders in the Sierra Norte de Puebla in Mexico and has now been rescued from nahua memory to cope with the COVID-19 pandemic as a food security strategy in the face of virus containment measures.

### Food, memory, and identity in two indigenous societies of Puebla in the context of the COVID-19 pandemic: a case study

According to [59], the global food system depends on 30 cultivated plant species for its functioning, and three of these crops feed more than half of the world's population [60]. This dangerous reduction in species and their varieties is accentuated by the control of seeds by a minority of transnational corporations that generate increasingly specialized varieties without adaptive genetic plasticity [61–63]. Recent studies have pointed to the importance of incorporating new wild cultivars into the human diet to ensure food security [64, 65].

A small region such as the Sierra Norte de Puebla has 182 species of edible plants, which are collectively used and distributed through local markets in the municipalities that make up the region [66, 67]. According to [20], the genera *Amaranthus, Begonia, Cucurbita, Brassica, Sechium, Peperomia, Rumex, Solanum, Porophyllum, Phaseolus and Nopalea* are identified in the region (*see* supplementary data 1). There are other species that are not traded in local markets, these are exchanged by the indigenous people. All edible plants are consumed fresh; the forms of preparation are varied and include rudimentary methods of transformation to increase their digestibility and palatability, as well as to eliminate toxic substances.

Most edible plants come from the indigenous food system, i.e. from home gardens, family plots or milpas (symbiotically sustainable crop associations, e.g. maize-beans-squash). Many species are wild, and others are cultivated, but their use and management imply a process of humanization. The ancestral knowledge that the indigenous people of the Sierra Norte de Puebla have is a strategic advantage; for example, the management of the COVID-19 pandemic in the region has forced the indigenous people to make use of their edible plants that provide food of nutritional value. Disruptions in the local economy have dynamized the ethnic diet in the region; the changes combine economy and health, although they obtain a complementary economic income from sales in the local market, the consumption of their plants provides the healthy diet necessary to combat the pandemic.

The COVID-19 pandemic has taken humanity by surprise, the rapid spread of the virus has left several sectors of the economy (e.g. tourism, travel, food system) in shock [68, 69]. As of 16 February 2022, WHO has reported 412 million people infected and 5.8 million people dead from the COVID-19 pandemic worldwide [70].

COVID-19 threatens global food security, especially in developing countries [71]. The current debate on food security calls for the construction of a research agenda that responds to the goals of the 2030 Agenda and transforms food systems without overstepping the boundaries of sustainability [72]. According to [73] global governance met in Paris in September 2015 under the leadership of the United Nations and agreed on the 2030 Agenda. The 2030 Agenda comprises 17 sustainable development goals and 169 sub-goals that serve as a global benchmark for the transition to sustainability. The agenda recognizes that different issues such as poverty, hunger, health, education, gender equality, environmental degradation, among others, are intertwined and can therefore only be addressed in synergy.

The indigenous food systems of the Sierra Norte de Puebla are based on a socioecological reality that has been historically constructed since ancestral times. According to Guzman [74], this means that food is determined not only by the environment but also by the values and meaning attributed to food.

These food systems have historically been differentiated from the non-indigenous population, although they are increasingly integrated and altered by the latter [74]. The recovery of ethnic biocultural memory is not only strategic for future generations of indigenous populations but can also form the basis for non-indigenous food-poor territories to configure new food systems. In this logic, an alternative is proposed that allow for a transition towards the sustainability of food systems, and the proposal focuses on rescuing the food preserved by indigenous groups through biocultural food memory. A diet derived from edible plants is nutritious and makes it possible to respond to the challenges posed by the COVID-19 pandemic.

In the two indigenous societies, the totonac and nahua, the role of women was relevant, the testimonies collected during the fieldwork show that the management of food knowledge covers most of the phases, i.e. identification and management of edible plants, recipes, different forms of preparation and transmissibility of knowledge. According to Ardren [75], in the prehispanic period, indigenous women played a relevant role in the new world, particularly in food preparation. Crown [76] argues that the differences between diet (food actually consumed) and cuisine (cultural beliefs and practices related to food) should be seen separately since women have traditionally had the knowledge of cooking, changes in cultural values about food affect women much more than men.

According to archaeological evidence and ethnohistory, ritual foods were difficult to prepare, as they required specialized training or ingredients that men did not possess, the author suggests that women who held knowledge of such aspects of cooking were highly valued individuals [75–77].

## Materials and methods

### Setting and participants

The study was geographically located in the Sierra Norte de Puebla, which is made up of two of seven regions of the Puebla state in Mexico; administratively, it is home to 63 of the 217 municipalities. It is a region that has been in dispute for more than a millennium due to its natural resources [78].

This region is part of the physiographic province of the Sierra Madre Oriental, its relief is mountainous, and its altitudinal gradient is between 200 and 2000 m above sea level, which gives the region a varied climate. Its territories reflect the traces of human interventions, showing a mosaic of landscapes ranging from primary vegetation, maize plots, coffee plantations, pastures, and home gardens [20]. The region maintains a natural wealth that is sustainably exploited by the indigenous people (totonac, nahua, hñähñu, tepehua) [79]. According to [20], there are 182 species of edible plants in the Sierra Norte de Puebla.

**Fig. 2:**
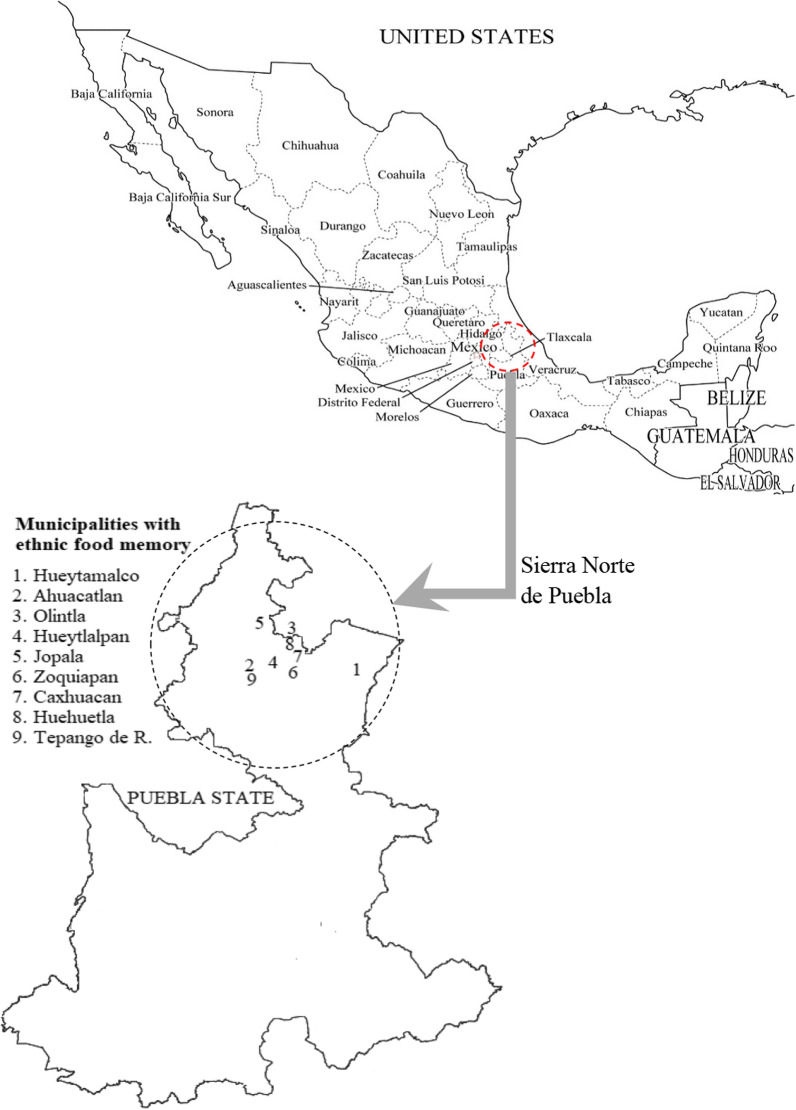
Location of municipalities with ethnic food memory

The study was conducted in nine municipalities of the Sierra Norte de Puebla, considering the following ethnic groups: totonac and nahua. Figure 2 shows the location of the participating municipalities. The research proposal was derived from a critical-reflexive analysis that started with the courses “environmental risks and climate change” and “society and nature” of the Educational Programme for Sustainable Development taught at the Intercultural University of Puebla State, in which students, in order to deepen the analysis of a topic, visited families and neighbours.

The fieldwork was conducted in November 2020 and May 2021, the study was based on a qualitative approach, using the semi-structured interview as a research technique. According to [80], semi-structured interviews are those in-depth interviews in which respondents have to answer pre-established open-ended questions. These types of interviews are conducted only once, with and individual or with a group, and usually cover the duration of 30 min to more than an hour. In a structured and organized way, with the help of a facilitator or moderator, a specific topic is explored.

This technique makes it possible to take into account the feelings, expressions, views, belief systems, values and traditions of the individual or group. The interviewer is able to follow thematic trajectories in the conversation that can deviate from the guide when deemed appropriate to re-learn the reality [81].

### Interview protocol

The interviews were carried out with heads of families with the following distribution: five families from nahua group settled in the municipalities of Hueytamalco, San Juan Ahuacatlan and Zoquiapan. In the totonac group, there were a total of eight families settled in the municipalities of Olintla, Hueytlalpan, Jopala, Caxhuacan, Huehuetla and Tepango de Rodriguez. A total of 13 heads of households were interviewed, of which eight were women between the ages of 40–80 years and five were men between the ages of 30–60 years. The dynamics of the interviews consisted of bringing the whole family together and the head of the family developed the topic through trigger questions with the support of other family members, establishing a reflective conversation around the history of their traditional foods, beliefs, values, food traditions and how the COVID-19 pandemic influenced the majority of foods that are derived primarily from wild plants.

As an important aspect during the interviews, the biocultural enquiry was unveiled by the heads of families through the oral tradition that has been passed down through generations in each ethnic group, rescuing from memory the ancestral food customs. One example was the first conquest suffered by the totonac ethnic group in prehispanic by the mexica who, due to their wars, required a constant supply of food, something that the totonac territories were able to provide.

### Historical trajectories of the interviewed ethnic groups

#### Totonac

The origin of the totonac ethnic group is uncertain [82], with the earliest records dating from 1259 to 1311. In the year 1450, there is a record of raids and conquest by the mexica [83]. Later records date from the Spanish conquest, from 1519 onwards, when surveys were designed to obtain information from the indigenous societies of the new world by mandate of King Felipe II of the Spanish Empire, these records were called “Relaciones”. In “La Relacion Geografica de Jonotla” dated between the years 1579–1581, it is stated that the first totonac passed through Teotihuacan, then to Atenamitie (Zacatlan), finally reaching the Sierra Norte de Puebla and Veracruz state.

The territories of totonac influence are mostly highly biodiverse, with irregular topography and a variable climate. These characteristics allowed for the development of adaptation strategies, for example, the use of natural resources through the different ecological levels of the Sierras (Puebla and Veracruz) (see http://pares.mcu.es/ParesBusquedas20/catalogo/show/304154?nm). This strategy allowed the totonac a constant supply of food in temporal terms and under a variety of climates, the advantages described above were the cause of the mexica conquest.

The memory of the ecological floors maintains this adaptation strategy in force and has allowed them to configure their food system, guaranteeing food outside the commercial circuits. With the arrival of the Spaniards in Veracruz in 1519, the totonac suffered a second conquest, the main cause of which was food. “Las Relaciones Geograficas” show the existence of two economic circuits in the region: the first of prehispanic origin and the second of colonial origin.

The system of beliefs, values and traditions forged in the prehispanic period progressively adopted elements of Spanish Christianity, but without disappearing, although the consequences for the indigenous people were fatal [84]. According to [85], the brutality and mistreatment of the indigenous population during the Spanish conquest added to the biological impact was devastating, the indigenous people had no antibodies or defences against the diseases brought by the Spaniards, generating epidemics that reduced the indigenous population from 11 to 3.7 million.

The ancestral totonac past in the context of illnesses has configured within the indigenous group a food system that contemplates health, an example of which is the widespread use of edible plants with medicinal properties. At present, the totonac are distributed mainly between the states of Puebla and Veracruz, with a total population of 438,756 indigenous people [86].

### Nahua

The nahua are the largest indigenous group in Mexico. However, it is not possible to speak of a single nahua group, as it is made up of diverse peoples who share the same language but who culturally present particularities, largely due to the environment they inhabit and the historical processes they have experienced [87].

The origins of the ethnic group date back to the end Preclassic period, when a village was built in the northeast of the Valley of Mexico that would become the largest and most imposing city in Mesoamerica. The hegemony that the nahua reached brought with it conquest, alliances, and trade routes, generating the expansion of prehispanic nahua power [88]. The period of greatness of the nahua came to an end with the arrival of Spanish in 1519.

The nahua peoples who entered the Valley of Mexico and nearby regions assimilated the doctrines and beliefs that were the legacy of the Toltecs. Among the consequences of these processes of contact are the different forms of syncretism that appeared in the religious thought of these peoples. The ancient worldview was preserved, but often interpreted in the light of new ideas [88].

At present, the nahua are mainly distributed among the states of Puebla, Durango, Nayarit, Distrito Federal, Estado de Mexico, Guerrero, Hidalgo, Jalisco, Michoacan, Morelos, San Luis Potosi, Tlaxcala, and Veracruz, with a total population 2.88 million people [86].

### Ethics

The study was carried out with respect for the traditions of the indigenous people, and permission was previously requested from the municipal authorities and indigenous communities, who gave their approval. The committee that endorsed and approved el study was formed by the university authority represented by the rector Guillermo Garrido Cruz.

### Data processing

The information obtained from the semi-structured interviews was systematized and analysed three categories; the first associated with the history of traditional foods, the second focused on the system of beliefs, values, and traditions and how this system contributed to the configuration of the foods, and finally, the experiences in the context of the COVID-19 pandemic and the response strategies in the face of the challenge of the health crisis.

From a biocultural perspective, the development of the interviews incorporated the importance of edible plants in the context of the COVID-19 pandemic. Indigenous elders also highlighted the importance of traditional foods, a story that was passed down to them orally by their ancestors. The data provided allowed for the construction of the trajectory of traditional foods mentioned in this study.

## Discussion of results

A total of 13 semi-structured interviews were conducted with a duration of one hour per interview in nine municipalities of Sierra Norte de Puebla. The application of the interviews rested in two ethnic groups in the region; the first group corresponded to the nahua ethnic group, in these five interviews were applied. The second group corresponded to the totonac, in the group eight interviews were applied. Although the interviews were conducted in a family setting, it was the heads of families who answered most of questions, other family members told anecdotal stories and gave some testimonies.

The heads of households were mostly women (61%) with ages between 40 and 80 years old, the women showed their knowledge about traditional foods, their ways of preparation and their relationship with their system of beliefs, values, and traditions (*see* supplementary data 2). This showed the close relationship that has been forged between the cultures of each ethnic group and nature. In a biocultural context, the heads of households aged between 70 and 80 told stories about edible plants and their usefulness when they were children and how their parents taught them this knowledge orally, which was remembered when the COVID-19 pandemic began (see Table 1).

**Table 1:** Main topic addressed during the interviews. The trigger questions were especially taken up and discussed by indigenous women from the Totonac and Nahua groups

Trigger questions in interviews	Totonac woman (*n* = *5*)	Nahua woman (*n* = *3*)	Totonac man (*n* = *3*)	Nahua man (*n* = *2*)
History	Transmission of local knowledge	Culture-nature relationship in ethnicity	Traditional medicine and the use of wild plants	Ethnic nursing treatments
System of belief, values, and traditions	The community's past	Worldview in the context of edible plants	Deities in the ethnic group	Community in the past and present
Traditional foods	Identification of edible plants and their preparation	Rescue of biocultural memory and ceremonial foods	Location and management of edible plants	Management of edible plants
Context in pandemic COVID-19	Revaluing the territory	Plants associated with nutrition and health	Revalorization of the biocultural food memory	Food memory in the ethnic group

The role of indigenous women associated with specialized knowledge of traditional foods takes on significance in the context of the Sierra Norte de Puebla, the division of labour in the region marking this trend. The literature reports the importance of indigenous women in prehispanic times and in the new world, particularly in food preparation [75, 76].

The research methodology made it possible to explore foods and edible plants that were forgotten at one point in history but have now been rescued from the biocultural food memory of the indigenous groups settled in the Sierra Norte de Puebla. The study identified two indigenous group, totonac and nahua, who have committed themselves to rescuing the memory of ethnic foods. This process of activation has been due to the health measures (e.g. confinement and spatial mobility restrictions) of the COVID-19 pandemic implemented in the region. Table 2 shows a mapping of ethnic foods, identified through the biocultural memory of the totonac and nahua indigenous people of the Sierra Norte de Puebla.

**Table 2:** Mapping of ethnic foods in the Sierra Norte de Puebla. Some foods, as well as several wild edible plants were particularly important during the COVID-19 pandemic in the region

Ethnic food	Municipality	Ethnicity	Method of preparation	Observations
Banana-tortilla	Hueytamalco	Nahua	Two ingredients are used in its preparation: green banana and maize dough*	The banana-tortilla is an adaptation strategy for the municipality. It is rescued in situations of vulnerability, such as the COVID-19 pandemic
Mafafa	San Juan Ahuacatlán	Nahua	En its preparation avocado, sesame or epazote leaves are used	This edible plant was rescued by the municipality's elderly due to the COVID-19 pandemic
Kilhxtik	Olintla	Totonac	Two ingredients are used in its preparation: Kilhxtik leaves and maize dough	This edible plant was considered a weed with the COVID-19 pandemic and was rescued and is now used to make tortillas and tamales
Tapixnuna Xkutna	Olintla	Totonac	With this plant, an ethnic sauce is elaborated that takes in its preparation: pieces of Tapixnuna Xkutna, chili, garlic and onion	Edible plant that due to the sanitary measures of the COVID-19 pandemic replaced the tomato
Potro ma´anta	Hueytlalpan	Totonac	Two ingredients are used in its preparation: donkey sweet potato bulb and chayote	Edible plant that due to the sanitary measures of the COVID-19 pandemic, its consumption was intensified
Quelite de pisis	Jópala	Totonac	In this edible plant, two parts are consumed: leaf and bulb. The method of preparation is boiled	Edible plant that due to the sanitary measures of the COVID-19 pandemic was rescued for consumption
Makuil kilit	Zoquiapan	Nahua	In this edible plant, the leaves are consumed, which are boiled	Edible plant that due to the COVID-19 pandemic, its consumption was intensified
Tatanchichi	Caxhuacan	Totonac	In this edible plant only, the fruit is consumed	Edible plant that due to the COVID-19 pandemic was rescued for consumption
Chayote	Huehuetla	Totonac	It is a vegetable that is consumed boiled	Vegetable that due to the COVID-19 pandemic its consumption was intensified
Xakg Xanan Stapu	Tepango de Rodriguez	Totonac	The feeding strategy has a double purpose to make use of the bean and the flower	Beans are consumed daily by the population, but due to the COVID-19 pandemic, the flower was also rescued for consumption
Malla Capen	Caxhuacan	Totonac	It is a legume, the pods are collected, dried and then roasted and ground. It is a substitute for coffee	This bean is used as a substitute for coffee, its consumption has intensified due to sanitary measures of the COVID-19 pandemic
Pulak'la	Tepango de Rodriguez	Totonac	The main ingredients are beans, chayote leaves, chili, sesame, banana leaves and maize dough	The consumption of this traditional food is historical, and the pandemic has intensified its preparation in the municipality

Maize-tortilla is made by a process of cooking native grain varieties with lime (nixtamalization) and cooking the tortilla when eaten, hot and fresh [89]

The totonac and nahua indigenous groups of the Sierra Norte de Puebla are historical occupants of the region and have built up ancestral knowledge of its food dynamics. Table 2 shows the different species of edible plants and their forms of preparation, rescued from the memory of the totonac and nahua peoples. Some eating habits have been modified by changes in the cultural logic influenced by globalization [90], such as the changes adopted in the preparation of Chayote (*Sechium edule*) or Xmala maklhtutkun (totonac name), which have added sesame seeds (*Sesamum indicum*) and tomato (*Solanum lycopersicum*) to their preparation.

**Fig. 3:**
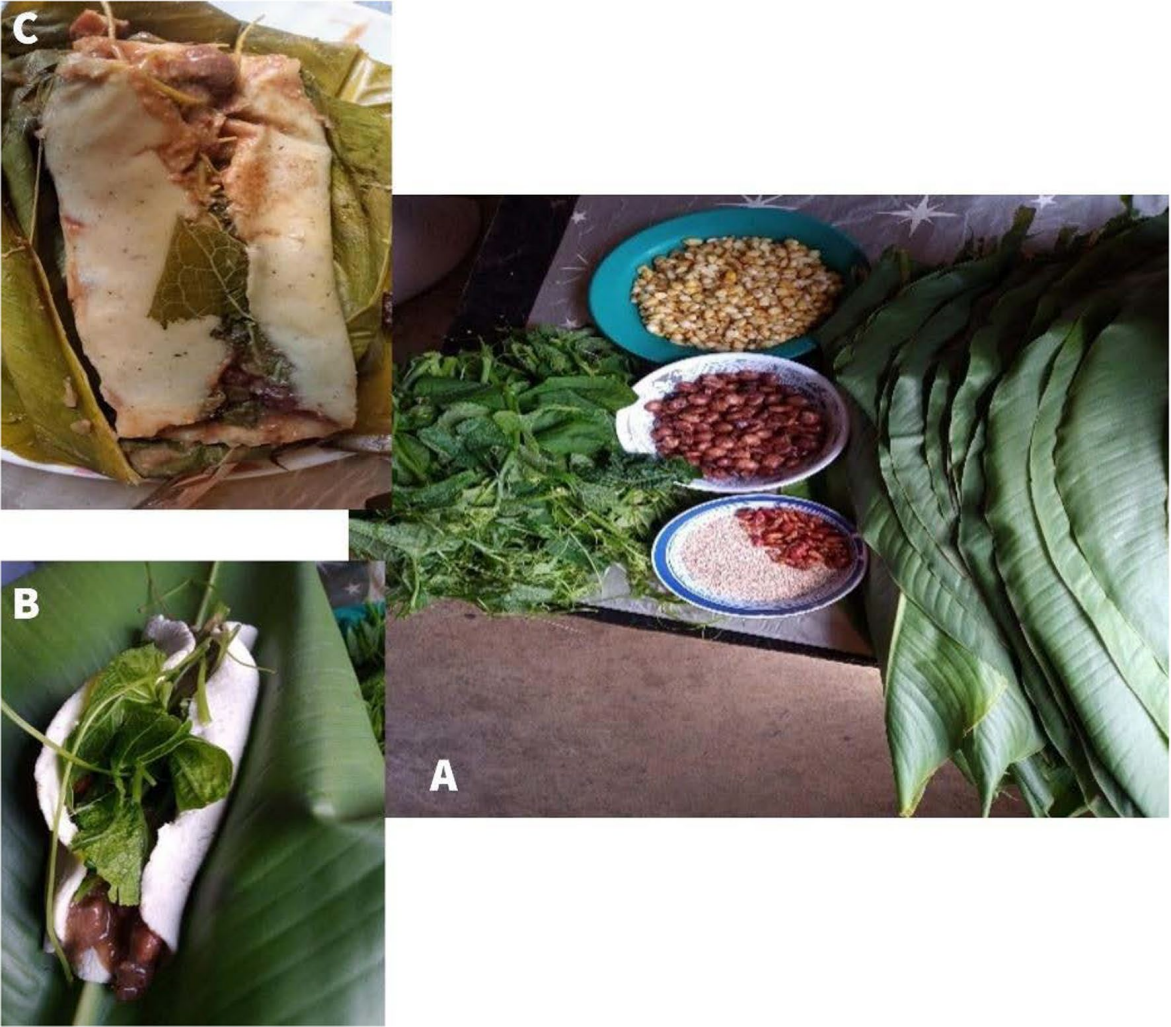
Traditional food "Pulak'la". **A** Ingredients: beans, chayote leaves, chili, sesame seeds, banana leaves, **B** How to eat it with maize-tortilla and **C** How to eat it with maize-dough

Another example where the added of external cultural elements is observed is the traditional food “Tamal de frijol” or “Pulak´la” (totonac name). It is prepared with beans, chayote leaves, chili, sesame seeds, banana leaves, and maize-dough/maize-tortilla (*see* Fig. 3). This food has been important during the COVID-19 pandemic as it is nutritious and allow them to strengthen their immune system because of the minerals and vitamins present in the traditional food.

Many plants still persist in the memory of indigenous people, as evidenced by the fact that they have been rescued from ethnic memory to be put at the service of indigenous people affected by the COVID-19 pandemic. The testimonies of the totonac and nahua indigenous people show this dynamic and its importance.

Ms. Avélina says that long ago in her community, because of poverty, they had to make maize-tortillas work. The way they made them work better was by mixing the nixtamal dough with green plantain dough. He told us that on one occasion his community suffered a strong storm that affected the crops, the river grew and took away most of the houses due to the landslides. Most of the neighbouring communities were cut off. Unable to get food for their consumption, they had to apply the method of making banana-tortillas. “...Now with the COVID-19 pandemic, once again we are forced to have more food because of the sanitary measures we must not leave our house...” (Mrs. Avélina, age 80, elderly indigenous Nahua woman, Community of Zompanico, Hueytamalco. November 12, 2020).

Ms. María mentions that when she was little her mother fed them a variety of quelites and that the cinco-quelites (Makuil kilit) were part of her almost daily diet, she liked to eat them a lot because her mother cooked them in different ways, at that time, in order for the ladies to fry their food, they used lard (oil substitute) because there was no bottled oil yet. However, the most common way to prepare it was just to boil it because butter was somewhat complicated to acquire and due to its economic condition, it was more viable to consume it in the same way it had been done before. He also talks about how with everything that is happening now regarding the pandemic, it would be feasible for the new generations to opt for consuming the quelites, especially Makuil kilit because it is very healthy, very tasty and can also be accompanied by some sauce in a molcajete and that like the quelite, its ingredients are obtained in the field or in the garden (Ms. María, age 86, elderly Nahua indigenous woman, Zoquiapan, November 13, 2020)

The interviews made it possible to identity three important traditional foods, which with the appearance of COVID-19 intensified their consumption, these being banana (*Musa acuminata* Colla x M. balbisiana Colla), beans (*Phaseolus vulgaris*) and tatanchichi (*Vasconcellea pubescens*). In the case of edible plants, nine species were identified, which have also been reported in the literature as useful plants lost importance in the diet of some indigenous groups over the years but remained in their biocultural memory. With the arrival of the COVID-19 pandemic and the ancestral knowledge about these plants, the totonac and nahua chose to rescue them for consumption. The edible plants identified are Malla Capen (*Macuna pruriens*), Xakg Xanan Stapu (Flower of the species: *P. vulgaris*), Chayote (*S. edule*), Makuil kilit (*Cyclantera dissecta*), Quelite de pisis (*Alocasia plumbea*), Potro ma´anta (*Dioscorea bulbifera*), Kilhxtik (*Tinantia erecta*), Tapixnuna Xkutna (*Begonia spp.)*, and Mafafa (*X. robustum*).

These edible plants provide the totonac and nahua diet with fibre, carbohydrates, vitamins, and minerals, as well as antioxidants [20, 91, 92]. Quelites, the name given to edible plants in the Sierra Norte de Puebla are plants that form part of the indigenous food system, the leaves are generally consumed, and their occurrence depends on the climate of the region, which has altitudes ranging from 200 to 2000 m above sea level.

Studies carried out in the region report some edible plants are mentioned in Table 2, used by the totonac and nahua ethnic groups. These plants have a diversity of uses (e.g. ceremonial, food or medicinal), some are wild, and others are cultivated [20, 93, 94].

**Fig. 4:**
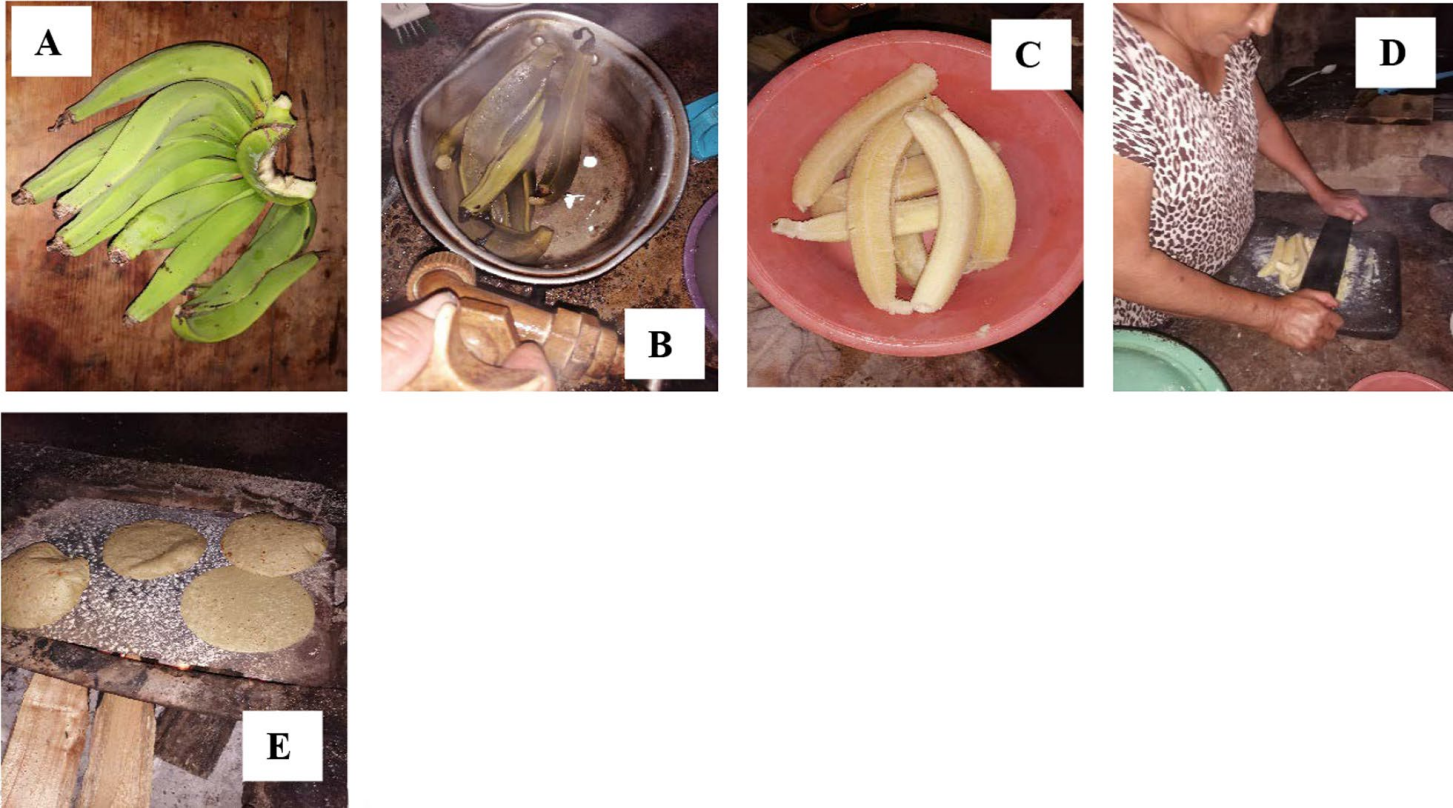
Sequence of the preparation of the banana-tortilla in the Community of Zompanico in the municipality of Hueytamalco, Puebla. **A** Green banana, **B** Cooked green banana, **C** Cooked green banana, **D** Preparation of banana dough (in equal proportions, maize dough is added), **E** Banana-tortillas are made on a metal plate and firewood. Banana-tortillas are the basis of food in the community, their preparation has become important because the community is in confinement due to the COVID-19 pandemic

**Fig. 5:**
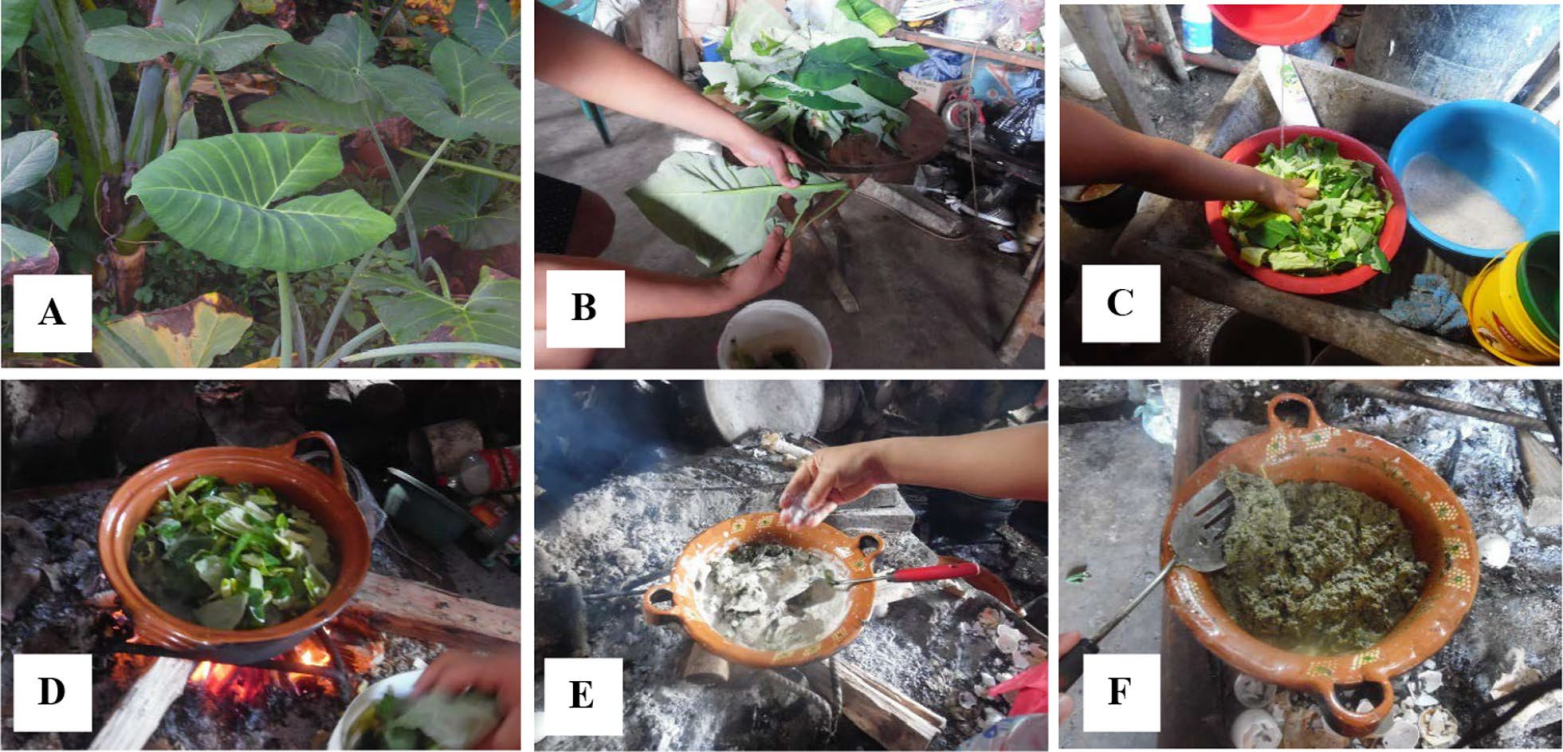
Preparation of the Mafafa. **A** Take the leaf from the Mafafa, **B** Remove the veins from the tender leaf so that it does not have a spicy flavour, **C** Wash the pieces of leaves, **D** Pour the leaves into a pot or container for cooking for 20 min. In the indigenous community there is the tradition of cooking with firewood, **E** The pot is removed from the first cooking process, the water is removed, and the following ingredients are added: avocado leaves *(Persea americana),* sesame seeds *(S. indicum),* cow-tongue leaves (*Rumex crispus*) y salt, **F** It is ready to eat, it is recommended to eat it with maize-tortillas

**Fig. 6:**
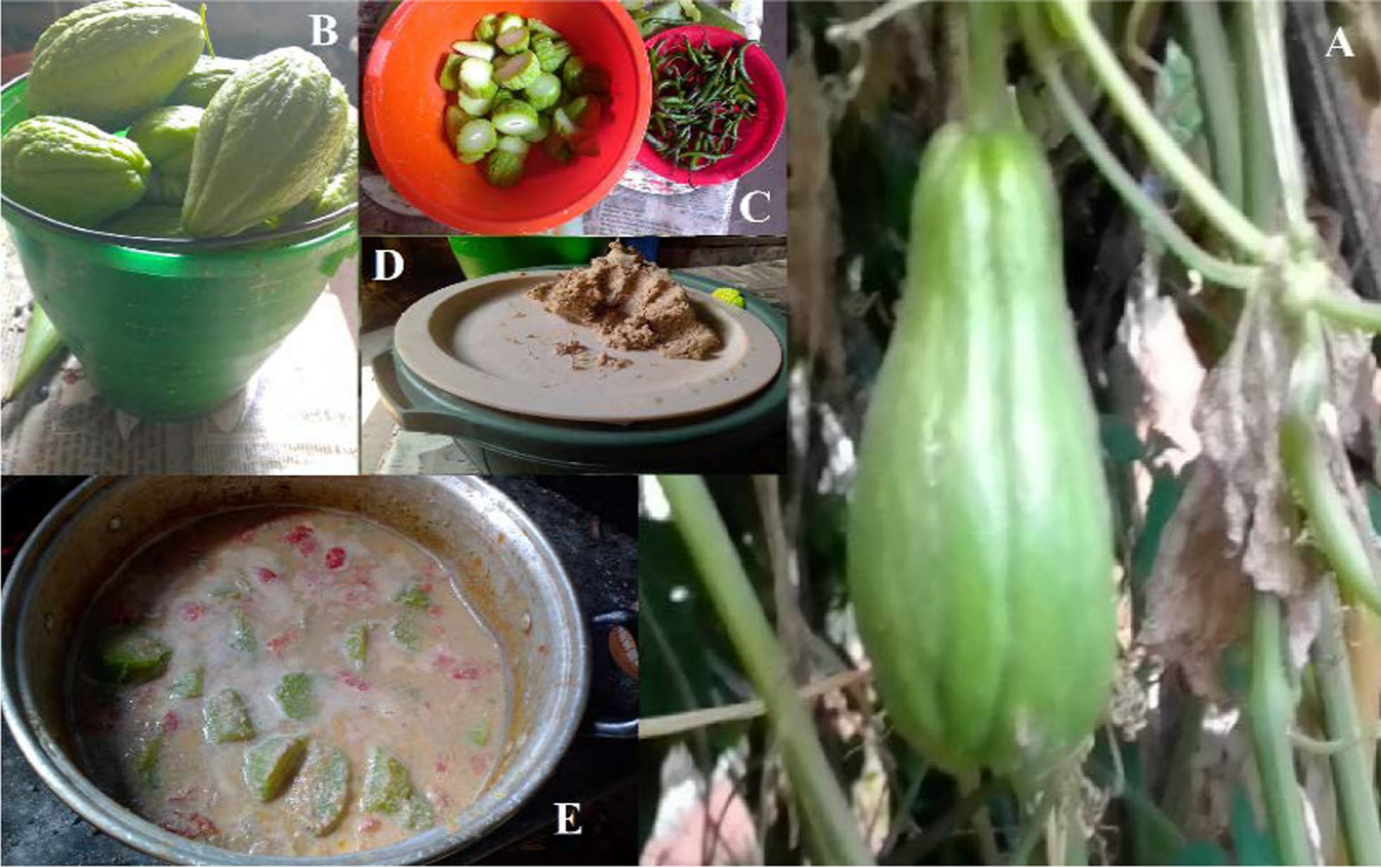
Preparation of the Xmala maklhtutkun. **A-B** Some chayotes (*S. edule*) are taken from the plant, **C** The chayote is cut in pieces, and it is joined with the following ingredients: sesame seeds (*S. indicum),* chili (*Capsicum annuum)* and tomato (*Solanum lycopersicum)*, **D** The sesame is toasted, then they are milled until obtaining a mass, then the chili and tomato are milled, and a sauce is obtained. In a pot with some water, we add the chayotes pieces, we put it to the fire (firewood). Once it boils, we add the sesame paste, chili and tomato. **E** Ready to eat

The preparation of quelites for consumption changes, they can be consumed directly, boiled, or fresh. Figures 4, 5, 6 show the preparation of some traditional foods.

**Fig. 7:**
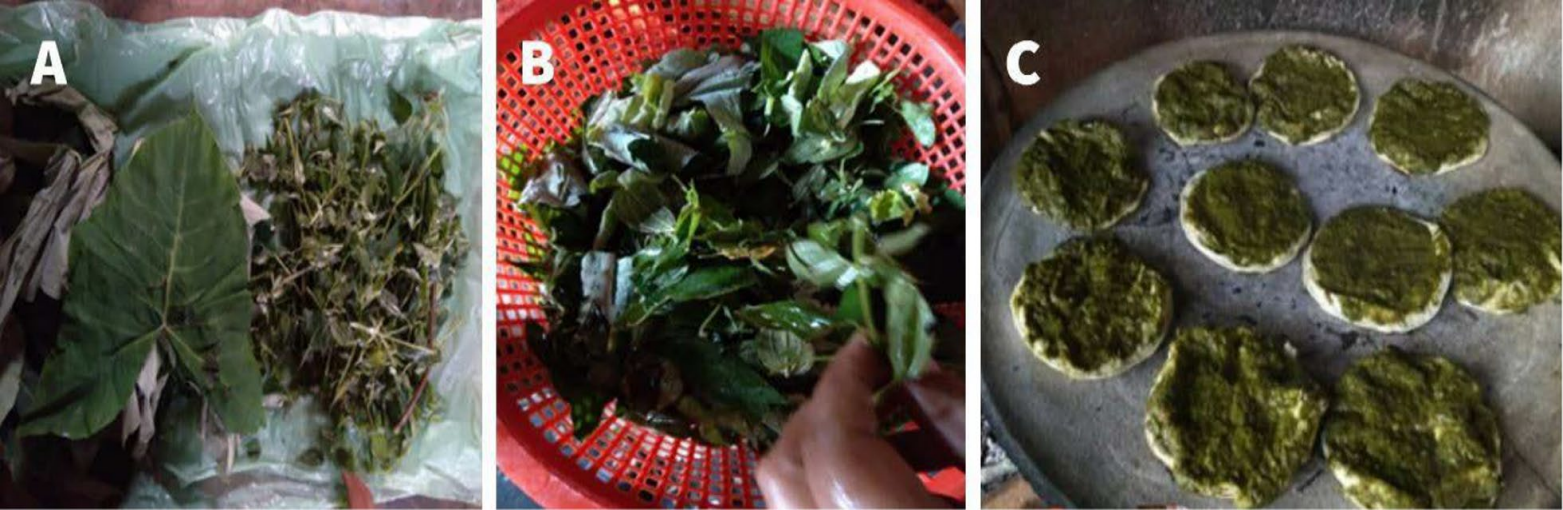
Another way of preparing the Mafafa. **A**,** B** Take Mafafa leaves and boil them, **C** Prepared a paste with the leaves, and add chili and sesame seeds. It is eaten by adding the paste to a maize-tortilla

Other ways of preparing Mafafa (*X. robustum*) were reported in the municipality of Caxhuacan, here the totonac make use of the leaves of Mafafa and a plant called Agrio, the leaves are put in hot water, when cooked they are mixed with Chili and sesame seeds, then ground to form a paste or cream. The paste is then combined with maize-tortilla (see Fig. 7).

Food memory is a coping strategy that has been practised for centuries by indigenous people, the experience of the indigenous groups of the Sierra Norte de Puebla being a case in point. Among the results of the analysis of the interviews, a revalorization of the consumption of edible plants to mitigate the effects (e.g. mobility, immunity) of the COVID-19 pandemic became visible. From a broader perspective, the deployment of this ancestral knowledge aligns with the current debate on mainstreaming sustainable food systems [65, 95] and shifts towards the consumption of healthier diets [29].

The indigenous people remembered the edible plants for their qualities and took advantage of the opportunity offered by the COVID-19 pandemic due to the sanitary measures established (confinement and restriction of spatial mobility) to rescue them. The families identified were: *Araceae, Begoniaceae, Cucurbitaceae, Commelinaceae, Caricaceae, Dioscoreaceae, Fabaceae, and Musaceae* (*see* supplementary data 1). The families of edible plants in the region are numerous, but only those that the indigenous people listed as having been rescued or intensified for consumption are mentioned.

The importance given to edible plants by indigenous women and men has become more relevant today due to the spatial mobility restrictions caused by COVID-19. The testimonies of the indigenous totonac show this reality.

Mr. Bonifacio and Ms. Carmen spoke to us about the food importance of the Mafafa. They commented that in rural areas it was already beginning to be noticed that quelites were being replaced with canned and packaged products, but with this pandemic, people are returning to quelites as food for all, especially Mafafa or Paxnikaka (totonac name). This plant was again a very common food for the grandfather and other people of this town, because of the pandemic, the grandfather cannot go out to the markets often to buy food because of the virus that is present, so the grandfather returned to the life of before, consuming plants that are found in the mountains. The most outstanding edible plant was the Paxnikaka, which is very easy to prepare and does not have many ingredients, but the food is very tasty (Mrs. Carmen and Mr. Bonifacio, Totonac indigenous, Olintla, 13 November 2020).

The sweet potatoes (Potro ma'anta) are eaten; here, they are not poisonous if they are not cooked well any problem even there are children or older people who eat it raw, we cook it in the fire tastes more tasty. They are eaten in two ways, the first is boiled with water, is accompanied by bananas or chayotes. The second consists of toasting them on the fire with firewood. There are people who prepare it in atole (a drink of prehispanic origin made with nixtamalized maize dough to which various ingredients are added to give it different flavours, properties, and consistencies), they boil them and then they take off the husk, squeeze them, add boiled water and sugar with this is the atole (Mr. Mateo, totonac indigenous, El Crucero Community, Hueytlalpan, 13 November 2020).

## Conclusion

The analysis explored the dynamic of food, memory, and identity of two ethnic groups in the Sierra Norte de Puebla. The topics addressed based on the methodological approach employed in the study were presented in the context of the COVID-19 pandemic. The fact that the pandemic was a disruptive element in the local economy of the ethnic groups interviewed led to the rescue of foods and edible plants for reuse. The literature reports at least 182 species of edible plants with high nutritional value that exist in the region. It was found that the totonac and nahua ethnic groups of the Sierra Norte de Puebla region in Mexico revalue three foods and nine edible plants.

The foods and plants identified have a food use, although some species of the same family may be found to have different uses (e.g. ceremonial, or medicinal). In the region, the whole plant is edible (leaves, stems, bulbs) and cooking methods may vary according to the qualities of the plant. The study supports the strategic importance of food memory for the totonac and nahua ethnic groups and shows the potential of food heritage for the rescue of plant species that have historically been part of their ethnic diet. Tracing ethnic foods is a revalorization strategy capable of transforming the way of conceiving consumption and food security in the face of the COVID-19 pandemic. From a broad perspective, it constitutes an alternative to conventional food systems that under a capitalist production model, promote food poverty in vulnerable territories.

The study showed the food, memories, and identity of only two ethnic groups in the region. The region has 63 municipalities where seven ethnic groups live, including the two ethnic groups under study. This suggests that food dynamics in the context of the COVID-19 pandemic may have a higher level of complexity. A research agenda oriented to deepen this type of study would contribute to the design of public policies in the field of mitigating food insecurity in Mexico as well as in other regions of the world.

## Supplementary Information

**Additional file 1.** Some families of the species of importance to the totonac and nahua ethnic group in the Sierra Norte de Puebla.

**Additional file 2.** Degree of importance of the answers given by the heads of households. Note: Categories of importance: Under = 1; Medium = 2; High = 3; Very high = 4.

## Data Availability

All data and materials have been presented in the paper.
