# Supplementary figures and images for: Looking into the past to build the future: food, memory, and identity in the indigenous societies of Puebla, Mexico
Source: J. Ethn. Food. 2022 Feb 23;9(1):7. doi: 10.1186/s42779-022-00123-w (PMC8864605; doi:10.1186/s42779-022-00123-w)

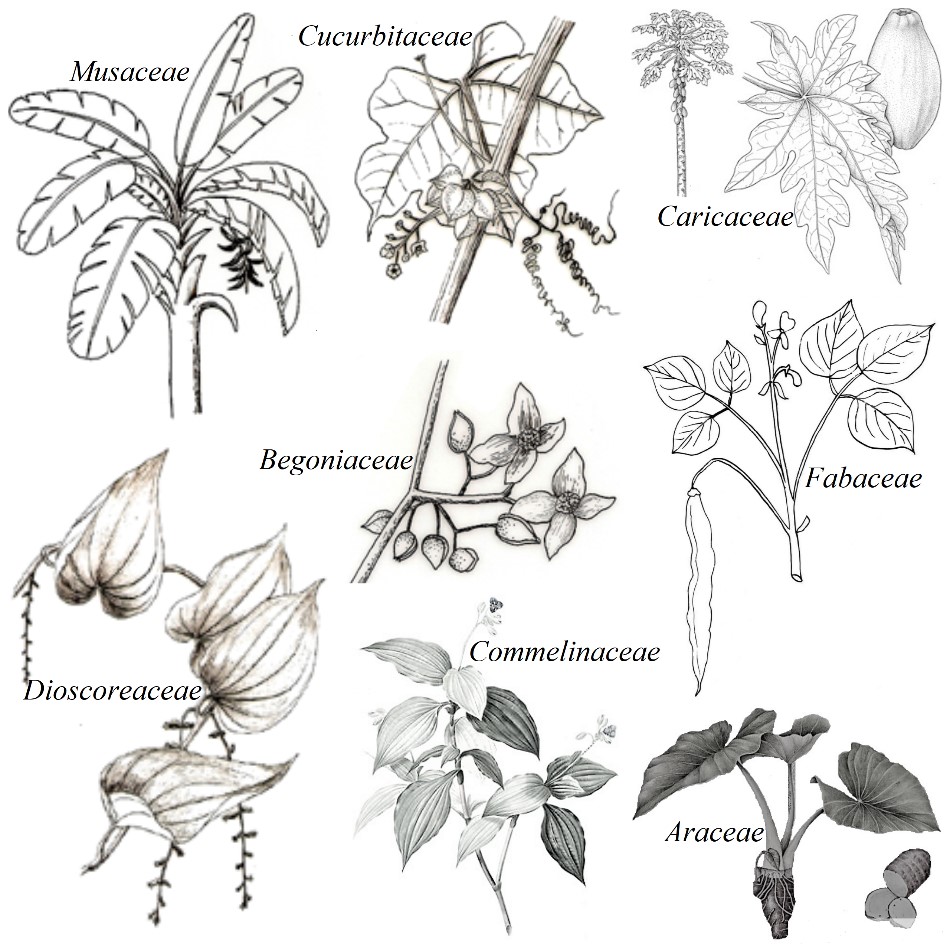

Supplement: Supplementary file 1 — Additional file 1. Some families of the species of importance to the totonac and nahua ethnic group in the Sierra Norte de Puebla. [file 42779_2022_123_MOESM1_ESM.jpg]
